# “I don’t hesitate to use the left-over antibiotics for my child” practices and experiences with antibiotic use among caregivers of paediatric patients at Zomba central hospital in Malawi

**DOI:** 10.1186/s12887-022-03528-3

**Published:** 2022-08-03

**Authors:** Redson Biswick Machongo, Alinane Linda Nyondo Mipando

**Affiliations:** 1 Department of Pathology, School of Medicine and Oral Health, Kamuzu University of Health Sciences, Private Bag 360, Blantyre, Malawi; 2 Department of Health Systems and Policy, School of Global and Public Health, Kamuzu University of Health Sciences, Private Bag 360, Blantyre, Malawi; 3 Kamuzu University of Health Sciences, Private Bag 360, Blantyre, Malawi

**Keywords:** Antibiotic resistance, Antimicrobial resistance, Caregivers, Paediatrics, Antibiotics

## Abstract

**Background:**

Inappropriate use of antibiotics is among the major causes of the global emergency of antibiotic resistance among children. The problem of inappropriate use of antibiotics among children is of special concern because they are still developing immunological systems, hence they are susceptible to many infectious diseases. As such, they receive a considerable disproportional amount of antibiotics which exposes them to antibiotic resistance. This study explored the lived experiences of caregivers of children under the age of five years on the use of antibiotics at Zomba central hospital.

**Objective:**

The main aim of this study was to explore the lived experiences of caregivers of children under the age of five years on antibiotic usage at Zomba Central Hospital, Zomba-Malawi.

**Methodology:**

This was a descriptive qualitative study with a phenomenological approach to explore the lived experience of caregivers of paediatric patients on antibiotic usage from May 2019 to July 2020. The study used interview guides to conduct in-depth interviews with 16 caregivers and purposive sampling was used to select the participants from the children’s ward. All interviews were audio-recorded and qualitative data was transcribed verbatim and thematically analysed manually to extract major themes and concepts on the subject matter.

**Results:**

Caregivers had little knowledge about antibiotic use and its resistance. most caregivers use the antibiotics inappropriately through self-medication, use of left-over antibiotics, buying antibiotics without prescription, and sharing of antibiotics.

**Conclusion:**

Based on the findings of this study, investment in public awareness and organising community-led interventions in antibiotic use related information is key to improve the quality use of antibiotics. The Government should focus on promoting interventions that lessen the indiscriminate use of antibiotics among the caregivers. Stringent laws need to be enforced by the government to restrict the access of antibiotics to parents without a prescription.

## Introduction

Antibiotic resistance in children is a global public health issue that is increasing and has implications for morbidity, mortality, and health care cost both in hospitals and in the community [1]. The spread of antibiotic resistance is causing not only increased morbidity and mortality among children but also a high economic burden by increasing the cost of health care due to prolonged stay in the hospital, additional diagnostic investigations, and the use of more expensive medications [2, 3]. Inappropriate use of antibiotics among children is of special concern in low- and middle-income countries because of the higher prevalence of infectious diseases and shortcomings in hygiene, sanitation, and public health in these contexts [3]. In addition, it is more dangerous to children’s health status because they are still developing immunological systems, hence they are susceptible to many infectious diseases, as such they receive a considerable disproportional amount of antibiotics which exposes them to antibiotic resistance [4, 5]. Inappropriate use of antibiotics encompasses prescribing against clinical guidelines [5] which includes over-prescription by physicians (such as for viral infections) [6]; inappropriate self-medication [5] which is aggravated by easy access to antibiotics for self-medication [7] and improper use of antibiotics such as overuse, underuse, and misuse [5] which is partially influenced by parents limited knowledge about antibiotics [7].

The overuse of antibiotics for treating upper respiratory infections among children is common regardless of geographic area, payment source, patient demographics, and physician specialty [7, 8]. In the African region, inappropriate use of antibiotics is largely in the form of non-prescription sales with almost 80% of people visiting drug outlets before seeing a health worker when they fall ill [9–11]. In Malawi, the inappropriate use of antibiotics has been due to the availability of antibiotics without prescriptions [12, 13], lack of knowledge, and the behaviour of consumers, and providers of antibiotics [14]. Parents’ perceptions and practice of how to use medicine have important effects on the management of childhood illness [15].

Health Belief Model (HBM) was used in this study as a conceptual framework because, the concepts and relationships described within the HBM work synergistically to create a greater understanding of the phenomenon of interest, reducing or avoiding a disease condition, and an aim to explain or predict health behaviours [16]. Therefore, to determine the use of antibiotics among children under the age of five years, we conducted a descriptive qualitative study with a phenomenological approach using the HBM tenets explore explore the lived experiences of caregivers of children under the age of five years on the use of antibiotics at Zomba Central Hospital in Malawi. The findings from this study will help to sensitize caregivers about the appropriate use of antibiotics and will be used to develop interventions and inform policymakers to formulate policies that will help to prevent the development of antibiotic resistance in Malawi.

## Methods

### Design

This was a descriptive qualitative study in the phenomenological tradition that emphasizes understanding the meanings people make of their experiences [17] and was conducted from May to June 2020. A qualitative method was chosen because of its usefulness in allowing the researcher to investigate social realities by exploring people’s experiences, behaviours, and opinions about the phenomenon [18].

### Research setting

The study was conducted at Zomba Central Hospital (ZCH) in the Children’s Ward. ZCH is a tertiary facility, and services six district hospitals in the south-eastern region of Malawi and 31 primary health care facilities within the Zomba district. The facility provides care and services to clients with different socio-economic, demographic, and educational backgrounds.

The admissions into the Children’s Ward in Zomba in 2019 were secondary to Malaria (20%), Pneumonia (18%), and Sepsis (10%). Out of 3,494 children reviewed by clinicians in 2019, 2, 818 received antibiotics such as Amoxicillin (80.6%), Erythromycin (9.8%), and Ciprofloxacin (7.6%), Doxycycline (2.0%) between the period of January to June 2019 [19].

### Recruitment, sampling, and sample size

Participants in this study were recruited using a purposive sampling approach and it allowed the inclusion of participants with rich information on the subject matter [20, 21]. Sixteen [16] participants were recruited for the study. The participants were of different demographic characteristics (Table 1). The sample size was deemed adequate following assertions by Mason that for all qualitative research, fifteen is the smallest acceptable sample size [22]. Furthermore, Guest et al. have argued that data saturation is often achieved by the 12th interview [18]. However, the study interviewed 16 participants and at this point, saturation was reached when no new information was generated from the participants.

The caregivers whose child was admitted to the children’s ward and was receiving antibiotics were identified with assistance from the Nurse-In charge. We purposively selected caregivers (CGs) that were 18 years of age and above, who had children on parenteral or oral antibiotics. Caregivers with under-five children in a critical condition were not recruited to participate in the study. All the participants willingly participated in the study and none refused to take part in the study.

### Data collection

The data were collected between May and June 2020, through In-depth interviews using an interview guide and a pictorial diary of drug administration.The interview guides were developed and designed by the author based on the specific objectives of the guided study, guided by the Health Belief Model and after reviewing other questionnaires in earlier studies [23, 24]. The pictorial diary of drug administration depicted instruments such as teaspoons, dosing cups, and oral dosing syringes used to administer antibiotics. The data collection tools were piloted at Zomba Central Hospital to ascertain their ability to capture the desired information and the appropriateness of the questions in local settings. The interviews were conducted in English and Chichewa based on the preference of the participants. All sessions were recorded using a digital voice recorder and lasted about 25–30 min on average. We adapted four-dimension criteria to ensure rigor in our study and this comprised strategies such as building the research team; preparing data collection guidelines; defining and obtaining adequate participation; reaching data saturation and ensuring high levels of consistency [18]. We ensured the credibility of the findings through member checking which was done by summarizing key points at the end of the interview. Secondly, dependability was achieved by maintaining consistency in the process of data collection specifically by using the same main questions in the interview guide. Thirdly, conformability which aimed at ensuring that study findings are representative of the participants’ views was achieved by including excerpts from the participants’ narratives. Finally, the researchers provided a dense description of the research methodology and the research context as a measure of ensuring the transferability of findings [25].

### Data management and analysis

Data were analyzed manually following a thematic data analysis approach as suggested by Braun [21]. Audio data were transcribed and translated verbatim from Chichewa into English. A unique identification number was assigned to each recorded interview. The transcripts were read multiple times for data immersion and familiarization, and also to get meaningful segments and essence. During this process, notes and markings were made within the transcript for coding [21]. Two other independent authors proofread and coded the transcript. The different codes were discussed and agreed to establish a coding framework. Similar codes were grouped under an overarching theme. The generated themes were reviewed iteratively, to assess if they were relevant to be themes or sub-themes and this determination was based on the richness of the codes under the theme. Themes that did not have enough supporting data were separated and combined with other themes to form one theme [21]. Safety of the recorded audios was ensured by using a password-protected laptop with access limited only to the researcher. 

### The conceptual Framework of HBM

**Fig. 1:**
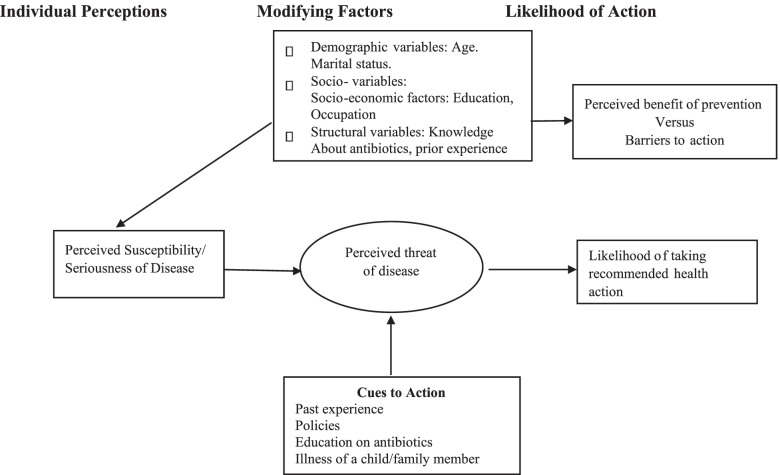
Conceptual Model of HBM. Adapted from Tarkang and Zotor [16]

This study used the HBM as a conceptual framework to analyse themes on experiences and practices of caregivers regarding antibiotic use. The HBM asserts that the motivation for people to take action to promote or prevent disease is based on the following concepts: perceived susceptibility, perceived severity, perceived benefits, perceived barriers, cues to action, and self-efficacy [16] as shown in (Fig. 1). The HBM is the most frequently used theory in health education, health promotion, and disease prevention [16], and health education programmes related to antibiotic use can be developed to better fit the needs of caregivers in antibiotic use. In this study, the HBM was used to deduce factors that influence caregivers of paediatric patients to use antibiotics.

### Ethical considerations

Ethical clearance was granted by the College of Medicine Research Ethics Committee (**COMREC P.01/20/2922**) while The Hospital Director offered institutional support. The matron and the nurse in charge of the paediatrics department were briefed on the aim and objectives of the study. Participants were informed of the purpose, significance, benefits, and risks of the study before their participation. Participants who agreed to the study provided written or thumb-printed informed consent and were free to stop the interview at any point during the study. Codes were used to identify participants to conceal their names for confidentiality. Interviews were conducted in a private room to achieve privacy. We carried out this study in accordance with relevant guidelines and regulations from the COMREC.

## Results

### Demographic characteristics of caregivers

The 16 caregivers that participated and were interviewed in the study were from Zomba District and were all females. Eleven caregivers attended up to primary education and only two caregivers attended tertiary education while three had a secondary school education (Table 1).

**Table 1:** Characteristics of caregivers, 16 women

Variable	Number
**Number of Children Alive**	
1	5
2	4
3	2
4	5
**Ability to read/write**	
Yes	13
Use of thumb	3
**Occupation**	
Formal employment	2
Not employed/ House wife	4
Self-employment/ Small Business	10
**Area of Residence**	
Rural areas	11
Urban areas	5

### Knowledge about antibiotics use and resistance

Most caregivers did not know about the name “antibiotics”, rather they were familiar with only a few examples of the antibiotics and the conditions which are treated by antibiotics. Familiar examples of the antibiotics were Bactrim, Amoxycillin, and Penicillin, including Flagyl (Metronidazole).

*“Yes, I know these drugs like amoxicillin, Bactrim, Penicillin, and Flagyl, because I have used them when my child was sick at different times, however, I did not know that they are called antibiotics. We just say it’s a medicine like any other”****Caregiver # 3.***

However, some caregivers referred to anti-malarial drugs, and pain killers as antibiotics, for example, Lumefantrine Artemether (LA), Fansidar (Sulfadoxine/Pyrimethamine), Panado, Brufen Aspirin, Cafinol, and Flagyl. They admitted to having used them many times as antibiotics. Some caregivers didn’t know the injectable antibiotics, such as Benzylpenicillin (X-pen), Gentamycin, and Ceftriaxone.

*“I know, these are malaria drugs, LA, Fansidar, Amoxicillin, Brufen, Panadol, Bactrim, and Flagyl. Those are the ones I know, but then they are some which are difficult to mention. The ones I know and I use at my house. I use them mostly when my child is sick, and also when I am sick. so, like when my child is sick. at the hospital, they use the injection ones, but I also know penicillin, used for sores”. -****Caregiver # 1.***

According to the caregivers’ antibiotics are used to treat cough, sore throat, and diarrhoea. Amoxycillin and Bactrim are mostly used to treat cough and sore throat whilst Penicillin is used to treat body aches and sore throat. Some caregivers know Bactrim is used for fever and malaria treatment while Flagyl is used to treat diarrhoea.

*“Bactrim is given as a substitute, for malaria drug when the child has fever and diarrhoea, whilst Amoxicillin is also given when the child is coughing and when the cough comes with fever” -****Caregiver #3***

The caregivers were aware of the administration time for antibiotics to children, for example, it was indicated that Amoxicillin and Flagyl are given three times a day whilst Bactrim is given twice a day. However, there were challenges with caregivers in knowing the duration of the said antibiotics to be given to children. It was also noted that the caregivers administer the antibiotics according to the HCWs instructions.

*“I give amoxicillin in the morning, afternoon, and evening, while Bactrim is given twice a day in the morning and the evening. Mostly I give the medication according to the advice of nurse and doctors, but sometimes if I forget I ask my neighbours to help me on how to give the drugs to my child”-****Caregiver # 9***

Despite having limited knowledge of antibiotic resistance (ABR), some caregivers reported that they are aware that at some point antibiotics can stop working if used inappropriately among children. This was highlighted when the caregivers said that nowadays amoxicillin is not treating cough as it used to be in the past.

*“The antibiotics can destroy the body, especially to the kids when the antibiotics are used inappropriately. I have once stopped giving my child the drugs because I saw that the antibiotics, I was giving my child, she responds slowly, so to give my child this antibiotic, after 3 days you see that my child has responded and got better yah”****- Caregiver # 3.***

Some of the caregivers were of the view that antibiotic resistance arises when caregivers don’t finish the dosage of antibiotics given at the hospital, when they share the drugs with others, and when they buy the drugs from the market without advice from the hospital workers because you might use drugs which are expired.

*“Usually, the side effects arise when you have been given drugs at the hospital for the child and you don’t finish the dosage or you share them with your friend and if you just buy the medicine at the market without the advice from the health care workers, because u can buy wrong or expired drugs-C****aregiver #13.***

### Sources of information on antibiotics use and resistance

The caregivers indicated that health care workers are the main source of information about antibiotics. However, some caregivers said that they know and learn about antibiotics from friends and relatives in their communities, whilst one caregiver said she learned about antibiotics from school and the media.

*“These drugs I got to know them from the hospital, like when I come here, they tell you that the way the child is feeling, we are going to give you these drugs, this type of drug, for example, amoxicillin if the child is coughing so you get to know that ooh! so this is the name of this drug”. -* ***Caregiver #1***

*“I knew these drugs when my baby was sick, and I went to the hospital to receive them and from school, I got to know them. From my neighbours also I got to know about the names of the antibiotics” -* ***Caregiver #3.***

### Practices of caregivers on antibiotic usage

#### Self-medication

Caregivers indulge in self-medication with antibiotics. The practice of self-medication facilitates indiscriminate use of antibiotics among the caregivers. The sources of antibiotics used in self-medication are; buying antibiotics without a prescription, using leftover antibiotics at home, and sharing the antibiotics with friends and relatives.

##### Buying antibiotics without prescription

The caregivers usually buy the antibiotics to administer to their children (self-medication) from the store (Hawker), a drug store, or a pharmacy without proper prescription and instructions from the health care workers.

*“Sometimes when my child is sick, I just go to the shop and buy the medication, because I know that this cough and sores will need Bactrim or amoxicillin so yea, I just buy what they have and give my child”-****Caregiver # 6.***

##### The use of left-over antibiotics

The caregivers use left-over antibiotics frequently at home. Usually, these are the remaining antibiotics from previous illnesses of their children. Sometimes they buy and keep them in readiness for a future illness since under-five children fall sick regularly. Mostly the left-over antibiotics are used for similar conditions that the children were treated for previously and in some instances, the caregivers use the antibiotics in minor and self-limiting conditions.

*“I keep antibiotics in my house to use when my child gets sick, the same antibiotics I get from the hospital or the ones I bought if left I make sure to keep them safe, so when my child falls ill, I just use the same. Mostly I use Bactrim and amoxicillin” -* ***Caregiver # 13.***

*“When my child falls sick suddenly and the condition is serious, I don’t hesitate to use the left-over antibiotics or if I don’t have, I rush to my neighbours to ask for drugs or buy. The hospital is far and sometimes there is no medicine there so it’s better to help your child before it’s too late”.****Caregiver #11***.

##### Sharing of antibiotics

Caregivers, get antibiotics from friends and relatives. However, some caregivers acknowledged that the tendency of buying and sharing antibiotics is not good since they end up sharing expired drugs or some drugs may not necessarily work for the condition of their child. Otherwise, this is done just to relieve the symptoms of the child and sometimes it’s more convenient than just rushing to the hospital with any illness, especially minor illnesses.

*“It’s not a secret, the antibiotics we share. For example, if your child is sick u reach out to your neighbour or your relative for any antibiotic, they have so that u can use it on your sick child. Yes, indeed I can agree that it’s a bad practice to share these antibiotics because you can get an antibiotic which can’t work on your child or maybe it’s expired or different from the condition”-****Caregiver # 12***

### Administration of antibiotics

Mostly, antibiotics are administered orally and parentally as prescribed, however, some caregivers use a small teaspoon whilst the HCWs use calibrated cups and syringes in the ward.

#### Instruments that are used to administer the antibiotics

**Fig. 2:**
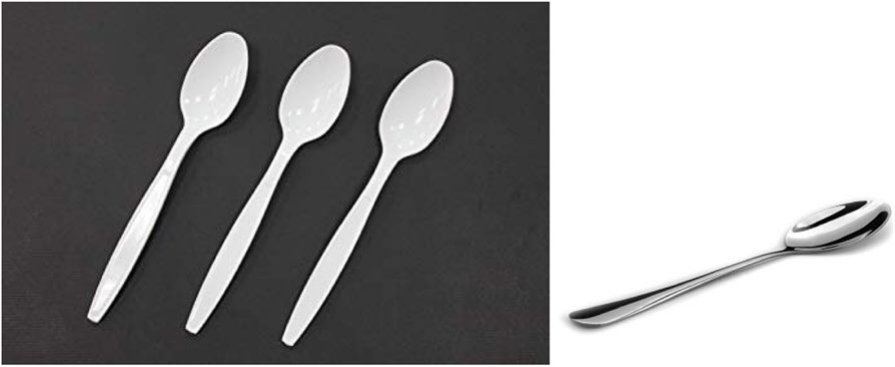
Pictorial diary, showing small teaspoons used by caregivers

The caregivers use a small teaspoon to administer the oral antibiotics in a tablet or a suspension form. Some caregivers emphasized that they give the correct dosage according to the HCWs instructions, whilst some of them were not sure if indeed the small spoon is equivalent to the dose to be given to the child (Fig. 2). However, some caregivers say it’s easy to administer using the teaspoon since some children cannot take and swallow the tablet and the suspended antibiotic by themselves.

*“I use the small teaspoon to give the antibiotics to my child. If it’s the tablets then I mix with water to dissolve them whilst for the suspension ones like amoxicillin syrup I just drop them on the teaspoon and give them. It’s very easy with the spoon since my child cannot swallow the tablets”-* ***Caregiver # 12.***

Some caregivers do not complete the prescribed antibiotic doses for their children at home. This usually occurs when they feel that the child’s condition has improved and they perceive that the child is fine and they conclude that there is no need to finish the treatment.

*“It happens that after giving the medication to the child there is a great improvement and now the child is fine and able to eat and play with friends. Now I see that at this point it’s not good to continue giving the medication because it might be too much to the body since the disease is now gone.”****Caregiver # 11.***

#### Storage of antibiotics

The caregivers store the antibiotics in a plastic bag in a cool place to avoid damaging them and they keep them away from their children. Caregivers said that they practice this to prevent water, and sunlight damage the antibiotics.

*“When I receive the drugs from the hospital, I put them in a plastic pack, and some in plain papers. when we get home, I transfer them to a plastic paper and store in a bag out of reach of children”-****Caregiver# 6***

## Discussion

The main findings from this study that explored the lived experiences of caregivers of children under the age of five years on the use of antibiotics at Zomba Central Hospital in Malawi were that there was limited knowledge and awareness about antibiotics among the caregivers. Self-medication with antibiotics was commonly practiced by the caregivers and they would buy antibiotics without prescription, use left-over antibiotics at home, and sharing of the antibiotics with friends and relatives. Most caregivers use a teaspoon in administering antibiotics and would store them in cool places.

The finding that most caregivers were not aware of the term “antibiotics” is contrary to findings in a study done in Taiwan where all subjects reported that they were familiar with the term” antibiotics”, but only 39.2% had correct knowledge about the use of antibiotics and 50.6% of subjects were unable to differentiate antibiotics from anti-inflammatory agents [26]. Our study results showed that the caregivers had difficulties identifying antibiotics and differentiating the antibiotic agents from other pharmaceutical agents. Similarly, studies that were done in Asia and Sub- Saharan Africa also showed that parents/mothers had incorrect or minimal knowledge about antibiotics and their use, and were unable to differentiate antibiotics from other anti-inflammatory agents [27–30].

There was a mixed understanding of the use of antibiotics which was similar to findings from an Indian study where the participants were confused regarding the conditions treated by antibiotics [13, 31, 32] with only a few participants aware that antibiotics are used against bacterial infection, while other parents (26.1%) in the same study thought that antibiotics are used against viruses and that antibiotics could be used for any microorganism [33]. Similar findings were reported in Nigeria where parents had a mixed understanding of the use of antibiotics [8, 34, 35].

Some of the parents in the earlier studies thought that antibiotics could cure infections caused by viruses, and some parents believed that antibiotics could shorten the duration of URTI symptoms, whilst some parents thought that taking antibiotics in advance could protect children from the common cold [24, 33, 36]. The confusion over the use of antibiotics on the effectiveness of antibiotics against bacteria and viruses is not a new finding and builds on previous studies [37]. The limited knowledge of antibiotics could be explained by the inadequacy of information on antibiotics in the public domain which is compounded by HCWs giving little or no instructions to caregivers about the antibiotics and their use. An earlier study done in Malawi has highlighted that the average consultation and dispensing time is too short to inform a patient about their medication adequately, possibly contributing to the confusion our participants had in distinguishing antibiotics from other categories of medicines [38]. In in Tanzania, a similar study argued that little/low knowledge on antibiotics may be attributed to the reason that during counseling, doctors use the general term ‘germs’ for indication of antibiotics, rather than specifically mentioning bacteria [10] thus people cannot differentiate between bacteria and viruses and hence concluding that antibiotics are effective against both [39]. Our findings revealed that most caregivers were aware of the time to give antibiotics and are consistent with a study in China where the participants demonstrated ambivalent attitudes towards compliance and completion of antimicrobial therapy and this discrepancy suggests that individuals would follow instructions on dosing regimens but not treatment duration [27]. Inadequate knowledge about the importance of completing antimicrobial therapy and awareness of antibiotics has been reported in several studies in Africa [27, 40]. We recommend that adequate information coupled with the right messages on the use of antibiotics and its consequences must be made aware to the public through the media, churches, and hospitals [41].

A systematic review explicitly specified educational messages regarding the appropriate use of antibiotics, such as the difference between viruses and bacteria and the specific illnesses for which antibiotics are indicated. Risks of inappropriate use, such as antibiotic resistance and potential side effects, were also commonly communicated [41].

As the HBM focus on psychosocial factors such as knowledge, attitudes, beliefs, intentions, and personality traits that influence behaviours [16]. It is imperative to design educational interventions that will influence the behaviours of caregivers on antibiotic use.

The fact that friends and relatives are a source of information about antibiotics necessitates mass campaigns so that communities are equipped with the right information on antibiotics to optimise the accuracy of information [27, 40, 42].

Although caregivers did not know about antibiotic resistance, they appreciated that antibiotics if used in every situation or in large amounts could be harmful which is consistent with previous studies where parents considered excessive antibiotic use could lead to antibiotic resistance [13, 14, 43, 44]. This implies that caregivers are likely to change their behaviours towards antibiotic use upon perceiving the severity of the consequences of inappropriate antibiotic use. This will promote optimal use of antibiotics among the paediatric population. There is a need to create an awareness of the term resistance to eliminate the confusion that may be there, particularly of what becomes resistant [32, 45].

Caregivers indulge in self-medication with antibiotics to their children which has the potential of resulting in applying an incorrect treatment [38]. Self-medication in children is common for conditions like colds and upper respiratory tract symptoms, which are self-limiting and mostly caused by viruses [46]. Notably, there is wide use of antibiotics among children under the age of five by their caregivers without seeking a physician’s advice because of the belief that the illness is mild and also draws the practice from previous experience with a doctor always prescribing the same antibiotics for similar conditions [38]. Women are confident in self-diagnosis and self-treating their children [10, 38] which necessitates targeted education for them since they are the primary caregivers (Table 1). In Malawi, just like most African countries, children spend more time with their mothers than with their fathers. This may explain why most women felt more confident in self-diagnosis and self-treating their children [12, 13, 47] These findings are in agreement with available published literature which indicated that intentions for self-medication with antibiotics result from the need to save money and the desire to act promptly to treat suspected or confirmed bacterial infections [13, 14]. Other studies in Africa have also reported higher self-medication rates for children by mothers [40, 48].

Contrary to our findings, other studies have reported a restrictive attitude toward self-medication among parents [35] with use only initiated after consultation with a doctor [13].

As it has been earlier reported, caregivers usually buy the antibiotics to administer to their children (self-medication) from the store (Hawker), a drug store, or a pharmacy without proper prescription and instructions from the health care workers [13, 42, 49].There is a need to strengthen the regulation over the sale of antibiotics. The use of left-over antibiotics to give to their children at home as reported in this study was reported earlier [6, 30] and parents stated that they were influenced by their social contacts in obtaining medicines, sharing antibiotics, or information on antibiotic use [13, 38]. The habit of sharing drugs is likely to result in people using the wrong treatment regimens and in sub-therapeutic doses [27]. This may introduce selection pressure and the development of resistance to antibiotics [50].

Despite teaspoons being the common tool used in administering antibiotics as was the case in our study and others [42], they are not calibrated to measure the actual doses which raises possibilities of over or underdosing. The capacity of household teaspoons ranges from 1.5 mL to 9 mL, potentially leading to errors in dosing [4]. We suggest the use of calibrated cups and droppers because they facilitate correct measurements. HCWs including Community pharmacists should educate caregivers on the selection and proper use of measuring devices to improve the accuracy of medication administration in the home [44, 51]. Our findings on the storage of antibiotics resonate with studies in Asia where the storage practice of antibiotics in a cool place and far from children is a measure of maintaining the potency of the drugs [52].

### Conclusion

The overall findings in this study found that there is limited knowledge regarding antibiotic use and resistance among the caregivers of children under the age of five years at Zomba central hospital. The study revealed that caregivers use antibiotics inappropriately through self-medication, buying antibiotics without prescription, use of left-over antibiotics, and sharing of antibiotics. Based on the findings of this study, investment in public awareness and organising community-led interventions in antibiotic-related information is key to improving the quality use of antibiotics. More interaction between HCWs and caregivers is needed to ensure that the caregivers receive adequate information and instructions related to antibiotics to improve the knowledge and practices among parents and thus, prevent inappropriate use of antibiotics that facilitate the development of antibiotic resistance. The government through the ministry of health should make deliberate Policies that focus on promoting interventions that lessen the indiscriminate use of antibiotics among the caregivers and the general public. Stringent laws need to be enforced by the government to restrict the access of antibiotics to parents without a prescription. The Government, through the MoH, must improve the accessibility of health facilities and ensure that resources are available so that people can visit the nearest hospitals with the hope of receiving quality medical care.

## Data Availability

The datasets used /or analysed during the current study are available from the corresponding author upon reasonable request.

## Abbreviations

AMR: Antimicrobial Resistance
ABR: Antibiotics Resistance
ABX: Antibiotics
HCW: Health Care Workers
CG: Caregiver
WHO: World Health Organisation
HBM: Health Belief Model
COMREC: College of Medicine Research Ethics Committee
UNIMA: University of Malawi
KUHeS: Kamuzu University of Health Sciences
KCN: Kamuzu College of Nursing
COM: College of Medicine
ZCH: Zomba Central Hospital
NOHRED: Norwegian Research and Capacity Building for Higher Education
